# Resistant and Apparently Resistant Hypertension in Peritoneally Dialyzed Patients

**DOI:** 10.3390/jcm14010218

**Published:** 2025-01-02

**Authors:** Bartosz Symonides, Marlena Kwiatkowska-Stawiarczyk, Jacek Lewandowski, Jacek Stanisław Małyszko, Jolanta Małyszko

**Affiliations:** 1 Department of Internal Medicine, Hypertension and Vascular Diseases, Medical University of Warsaw, 02-097 Warsaw, Poland; bartosz.symonides@wum.edu.pl (B.S.); jacek.lewandowski@wum.edu.pl (J.L.); 2 Department of Nephrology, Dialysis and Internal Diseases, Medical University of Warsaw, 02-097 Warsaw, Poland; marlena.kwiatko@gmail.com; 3 Transplantation and Internal Diseases with Dialysis Unit, Department of Nephrology, Medical University of Bialystok, 15-540 Białystok, Poland; jackmaly@poczta.onet.pl

**Keywords:** resistant hypertension, apparent treatment-resistant hypertension, end-stage renal disease, peritoneal dialysis

## Abstract

Hypertension in chronic kidney disease patients is very common. The definition of resistant hypertension in the general population is as follows: uncontrolled blood pressure (BP) on three or more hypotensive agents in adequate doses, or when patients are on four or more hypotensive agent categories irrespective of the BP control, with diuretics included in the therapy. However, these resistant hypertension definitions do not apply to the setting of end-stage kidney disease. True resistant hypertension is diagnosed when adherence to treatment and uncontrolled values of BP by ambulatory blood pressure measurement or home blood pressure measurement are confirmed. Due to these limitations, apparent treatment-resistant hypertension (ATRH) is now defined as an uncontrolled blood pressure on three or more antihypertensive medication classes or the introduction and use of four or more medications regardless of blood pressure level. Concerning dialysis patients, data are very limited on hypertension, its epidemiology, and the prevalence of apparent treatment-resistant hypertension in peritoneal dialysis. In this review, therefore, we discuss the hypertension definitions, targets of the therapy in patients on peritoneal dialyses, and their biases and limitations. We present the pathophysiology, diagnosis, and management of high blood pressure in the peritoneally dialyzed population together with published data on the apparent treatment-resistant hypertension prevalence in this population. Peritoneally dialyzed patients represent a unique population of dialyzed subjects; therefore, studies should be conducted on a larger population with a higher quality of drug adherence and target blood pressure values. The definition of resistant hypertension and apparent resistant hypertension in this group should be redefined, which should also consider residual kidney function in relation to both subclinical and clinical endpoints.

## 1. Introduction

Currently, there are three methods of kidney replacement therapy: peritoneal dialysis, hemodialysis, and kidney transplantation. Peritoneal dialysis (PD) also provides fluid removal together with solute clearance in end-stage kidney disease (ESKD); however, peritoneal dialysis utilizes the peritoneal membrane as equivalent to the dialyzer in hemodialysis.

The recent International Society of Nephrology Global Kidney Health Atlas (ISN-GKHA) presents data for the ISN Eastern and Central Europe region countries [1]. In these countries, HD was the predominant modality for kidney replacement therapy in patients with kidney failure; therefore, more data are available from hemodialysis patients. In this report, it is stressed that hypertension is the main reason for chronic kidney disease (CKD) and kidney failure. It is also emphasized that nearly one third of the patients in this part of Europe suffer from hypertension [2,3]. In patients on kidney replacement therapy, cardiovascular disease remains the leading cause of mortality with hypertension playing an important role [1]. On the other hand, the kidney could not only be the victim but also a culprit of hypertension [4]. As shown by a new retrospective observational study based on the Sicilian Registry of Nephrology, Dialysis, and Transplantation, hypertension in the participants could be a symptom of an unknown renal disease that causes ERSD in children, and a biopsy from these patients can reduce the misleading diagnosis in young patients [5].

In addition, in the dialysis population, hypertension is the most common finding; however, estimation of its prevalence varies substantially among studies due to differences in hypertension definition and its measurement methods, i.e., either before or after HD, or using ambulatory blood pressure (ABPM) data. It has been estimated that the prevalence of hypertension in hemodialysis (HD) patients reached as high as 85% and nearly 30% in peritoneally dialyzed subjects [6,7,8,9]; however, the data are relatively old with some dating to the last century [6,7,8]. In a cross-sectional study on Italian PD patients by Cocchi et al. [10] published in 1999, hypertension was prevalent in 88% of the patients. More importantly, 77% of the patients on hypotensive therapy had poorly controlled blood, leading to a high burden of hypertension. They used definitions from the World Health Organization/International Society of Hypertension and the Seventh Report of the Joint National Committee on Prevention, Detection, Evaluation, and Treatment of High Blood Pressure (JNC 7) criteria, which were valid at that time. In a more recent report from the American Hypertension Association, the prevalence of hypertension in the PD population was estimated at 29% to 80% [11].

Moreover, incident patients, i.e., those who are beginning dialysis, are more hypertensive than prevalent patients because they are more volume-overloaded, and time is required to achieve dry weight [12]. Although patients initially improved their blood pressure on peritoneal dialysis, later they tend to experience worsening blood pressure control due to volume expansion, which is in parallel with a further loss of residual renal function (RRF). Moreover, inadequate volume control despite the dialysis initiation often manifests as persistent hypertension [6,13]. As shown previously, Günal et al. [14] tested the hypothesis that blood pressure control could be achieved with a strong volume restriction without hypotensive drugs. In the majority of subjects on continuous ambulatory peritoneal dialysis (CAPD), they proved that blood pressure could be effectively controlled by severe salt restriction together with increased ultrafiltration by using hypertonic peritoneal dialysis solution containing 3.86% glucose. In their study, they were able to attain normotension in 91% of the patients with volemia control using UF and in 42% of the patients on strong salt restriction. However, data are very limited on fluid status and the relationship among fluid status, BP, and cardiovascular disease in the PD population.

As PD is a continuous technique with continuous fluid removal, it may be a preferred modality due to better preservation of residual renal function (RRF). In an older study, CAPD patients showed better blood pressure control [15]. Cnossen et al. [16] showed that control of both BP and volemia did not differ between APD and CAPD, although sodium removal was greater in CAPD.

## 2. Pathophysiology of Hypertension in Dialysis Patients

In the 1960s, when HD therapy was available for patients with end-stage kidney disease, Stokes et al. [17] postulated that in the majority of patients with ESKD, adequate ultrafiltration during HD together with restriction of diet sodium contributes to hypertension control in hemodialyzed patients. However, they also stressed that in some hemodialyzed patients, hypertension was uncontrollable.

Hypervolemia is the most important factor responsible for both systolic and diastolic hypertension among ESKD patients on HD [18,19,20] or PD [21].

PD, as a continuous modality of kidney replacement therapy, continuously removes water and uremic solutes, and the effectiveness of the removal determines the outcomes of PD patients [22]. As peritoneally dialyzed patients are not exposed to the hemodynamic effects of hemodialysis sessions, subclinical hypervolemia is more often found in PD.

On the other hand, PD patients may experience water-sodium retention due inefficient transport through peritoneal membrane and peritoneal injury, which contribute to poor control of hypertension [20,23]. In addition, in the setting of small or absent residual renal function, diuretics may not work [24]. The preservation of RRF is of major clinical benefit of PD. In a re-analysis of the CANUSA study, the importance of RRF was stressed as it was the parameter associated with survival, whereas neither net peritoneal ultrafiltration nor total fluid removal was linked to survival. [25].

Similar data on the beneficial effect of renal clearance on patient outcome and quality of life were later provided by the NECOSAD study [26]. However, Michels et al. [27] showed the RRF declined fast predominantly during the first 12 months of automated peritoneal dialysis (APD). These data lead to the assumption that RRF preservation is crucial to survival; however, in PD, striving for euvolemia while maintaining RRF is a trade-off. BP control is challenging with striving for RRF preservation at the price of volume excess.

In addition, sodium removal in PD is clinically relevant [28], as sodium accumulation in the interstitial tissue leads to the expansion of extracellular water, resulting in salt-sensitive hypertension [29]. In turn, it results in progressive RRF decline [29] and impacts negatively urinary sodium removal [30]. Recently, Bontic et al. [31] reported significant relations between sodium intake and total sodium removal and underlined the role of RRF not only in sodium removal but also in rise in lean body mass. On the basis of their findings, implementing PD prescription with maximal sodium removal is of the utmost importance, in particular in patients without RRF.

On the other hand, peritoneal transport may also affect blood pressure control [23]. Chronic intraperitoneal inflammation affects the rate of peritoneal solute transfer, which is then significantly associated with worse clinical outcomes in PD, i.e., mortality, technique failure, etc. [22,32]. In addition, peritonitis episodes contribute to intraperitoneal inflammation, causing higher peritoneal solute transfer rates and thus lower ultrafiltration [33], leading to hypervolemia and worsening of blood pressure control [33].

Moreover, long-term PD therapy leads to both structural (fibrosis, hyalinizing vasculopathy angiogenesis) and functional (increased rate of peritoneal solute transfer rate, failure of ultrafiltration failure) [34] changes and treatment discontinuation. APD and the use of icodextrin may affect the risks related to a fast rate of peritoneal solute transfer [22]. The fluid distribution in PD is likely different compared with HD [35]. In PD, hypoalbuminaemia is more common as a result of the additional peritoneal protein losses. It also reflects systemic and intraperitoneal inflammatory states [36]. On the other hand, intravascular plasma volume is typically normal in PD; however, in the case of hypoalbuminemia, excess fluid is mainly present in the interstitial compartment [37]. We also have to take into account that food and fluid intake is more liberal due to continuous treatment in PD. As patients are seen every 4–6 weeks for check-ups, non-compliance is an issue as well. As shown by Mehta et al. [38], non-adherence to hypotensive drugs is also a common cause of resistant hypertension in the dialyzed population. The Sharesource platform, used for the management of APD patients, may also help to cope with the problem of non-compliance by allowing treatment modifications [39].

Increased arterial stiffness is another important cause of systolic hypertension in patients with ESKD [18,19]. In 179 patients in the HDPAL trial (hypertension in hemodialysis treated with atenolol or lisinopril), the velocity of aortic pulse wave was independently associated with 44 h systolic blood pressure [40].

New-onset hypertension or the worsening of pre-existing hypertension is a common but frequently under-reported complication of therapy with erythropoietin-stimulating-agents, particularly in younger patients with diastolic hypertension [18,19,41]. 

Other factors involved in the pathogenesis of hypertension in ESKD are sodium overload, activation of the sympathetic nervous and renin–angiotensin–aldosterone systems and endothelial dysfunction [18,19,41]. 

**Figure 1:**
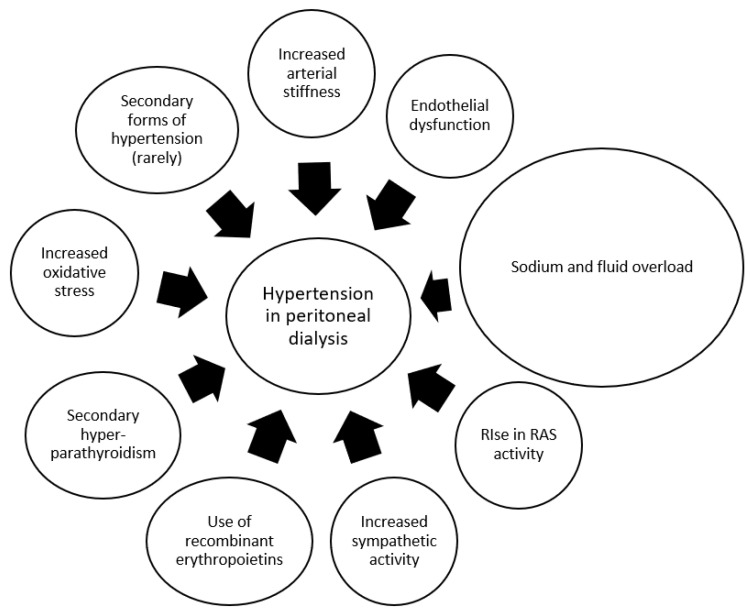
Potential mechanisms of arterial hypertension in peritoneal dialysis patients. RAS: renin angiotensin system.

The proposed pathophysiology of hypertension in peritoneally dialyzed patients is shown in Figure 1.

## 3. Hypertension as a Risk Factor in ESKD

The death rate in patients with ESKD in unadjusted analyses due to cardiovascular disease is 10–30 times higher, and it remains 5-fold higher adjusted for covariants [42]. The high mortality rate results are probably due to both a high case fatality rate and a high prevalence of cardiovascular disease [43]. In ESKD patients, mortality after major adverse cardiovascular events is highly elevated relative to the general population even after stratification for comorbidities such as diabetes [7]. U-shape relations between blood pressure and cardiovascular risk in patients with ESKD came from observational data [42,44,45]. Standardized office interdialytic measurements showed a linear association between systolic blood pressure in the range from 100 mmHg to 180 mmHg [46]. There are only a few observational studies in peritoneal dialysis patients assessing the relationship between BP and mortality. Udaray et al. [47] followed 2770 PD patients at 6 months from the start of dialysis in England and Wales in years 1997–2004 for 3.7 years (0.1–9.9 years range). They reported that greater systolic blood pressure, diastolic blood pressure, pulse pressure and mean arterial pressure were related to a lowered death rate in the first 12 months, but greater systolic blood pressure and pulse pressure were associated with an elevated late death rate (in years 6+) in fully adjusted analysis. They also underlined that the major limitation was a lack of blood pressure data for 3086 patients (excluded due to this reason) as well as no data on cardiac function or antihypertensive medication. Goldfarb-Rumyantzev et al. [48] studied associations between blood pressure and mortality in peritoneally dialyzed patients (n = 1053) from the USRDS prospective DMMS Wave 2. They found that systolic BP < 111 mmHg was related to greater death risk, whereas systolic BP of more than 120 mmHg was related to shorter length of stay at the hospital. In addition, subgroup analysis showed that this correlation existed only in subjects with diabetes and heart failure history who were treated with hypotensive drugs. They concluded that caution should be exercised regarding the administration of aggressive hypotensive therapy in PD patients. In one more study on the associations between blood pressure and mortality in PD, Afhsinnia et al. [49] evaluated 77 prevalent ESKD patients starting PD in 2007–2012, and the primary outcome was all-cause mortality. They reported that higher mortality was associated with lower BP, and this phenomenon could be mediated in part by worsening heart function. In the most recent study by Zhu et al. [50] on 1422 PD patients followed for a median of 26 months, a longer duration of apparently resistant hypertension (ATRH) in the first year of PD was associated with a higher cardiovascular death rate and worse long-term clinical outcomes.

## 4. Blood Pressure Assessment in ESKD

Although standardized blood pressure measurement is recommended in patients with chronic kidney disease by the current KDIGO guidelines on blood pressure, the specific recommendations for ESKD patients on dialysis are lacking [51]. Similarly, in KDIGO guidelines for chronic kidney disease, the problem of blood pressure control was put in the context of delaying CKD progression and management of its complications without touching ESKD. KDIGO recommended [2B level: level 2 means we suggest, and B means moderate certainty of evidence] that patients with high blood pressure and CKD should be managed to target systolic blood pressure lower than 120 mm Hg when tolerated using standardized office blood pressure assessment [52].

The use of ambulatory blood pressure measurement, ABPM in peritoneally dialyzed-PD patients was much less studied compared to hemodialyzed-HD patients with many small and underpowered studies [8]. The Italian Co-operative Peritoneal Dialysis Study revealed a 53% prevalence of non-dipper pattern among 504 PD patients [10]. In the study of 74 PD patients, ABPM correlated well with left ventricular hypertrophy in contrast to the office BP [53].

## 5. Resistant Hypertension in ESKD

The definition of resistant hypertension in the general population is as follows: uncontrolled blood pressure (BP) on three or more hypotensive agents in adequate doses or when patients on four or more hypotensive agent categories irrespective of the BP control with diuretics were included in the therapy [54,55]. However, this definition of resistant hypertension is not directly applicable to the ESKD setting [56]. True resistant hypertension is diagnosed when adherence to treatment and uncontrolled values of BP by ABPM-ambulatory blood pressure measurement or HBPM-home blood pressure measurement is confirmed [55].

Due to the fact that in many epidemiological studies, key elements such as medication doses, adherence to the therapy, etiology of HTN, and ABPM measurement for true resistant hypertension definition are missing, therefore, a proposition of apparent treatment-resistant hypertension (ATRH) as an additional category of HTN was introduced. The ATRH definition includes the term of an uncontrolled BP on three or more hypotensive drug classes, or the administration of four or more drugs irrespective of levels of BP [57]. Thus, ATRH applies to pseudo-resistant hypertension (i.e., white-coat hypertension, inaccurate measurements of BP, or non-adherence to drugs), controlled or controlled BP, and refractory hypertension (uncontrolled BP with at least five hypotensive drug classes) [54,58]. Apparent treatment-resistant hypertension (ATRH) prevalence in the recently published studies on PD patients is presented in Table 1.

In a cross-sectional study of 1789 PD patients from nine centers in Guangdong, China, Li et al. [59] assessed the prevalence of ATRH at 42.2% based on home BP measurements. Drug adherence was assessed in 15% of the patients by the Eight-item Morisky Medication Adherence Scale or pill counting. As many as 91.4% of the patients were classified as having uncontrolled resistant hypertension, which was defined as systolic/diastolic BP ≥ 130/80 mmHg while on treatment with three or more antihypertensive medications. The incidence of ATRH was associated with younger age, higher BMI (body mass index), decreased albumin and worse adequacy of dialysis. The authors did not count diuretics as a hypotensive drug class, pointing out the decreased effectiveness of these drugs in peritoneally dialyzed patients.

In 140 patients on PD, Vareta et al. [60] assessed the incidence of ATRH reaching 30% using the criteria of 130/80 mmHg for 24 h ABPM. A subclinical overhydration presence was assessed using the BIS (bioimpedance spectroscopy) method. Patients with ATRH were older and had a higher PD vintage, higher dialysate-to-plasma creatinine ratio, more frequent history of diabetes mellitus and were more often current smokers. The overhydration index in BIS was twice as high in ATRH. The volume overload (overhydration index in BIS > 2.5 L) had twofold higher prevalence in the ATRH group (38.1% vs. 18.4%), and it was a significant difference. The authors concluded that the achievement of adequate volume control may be a therapeutic opportunity to improve the management of hypertension in this high-risk patient population. In another paper from this group [61], the prevalence of office BP ≥140/90 mm Hg and ambulatory hypertension with BP ≥130/80 mm Hg was 92.9% and 95%, respectively. In addition, 31.4% had controlled hypertension, 5% had white-coat hypertension, 19.3% had masked hypertension, and 39.3% had sustained hypertension. Isolated nocturnal hypertension was detected in 23.6% of patients. Vaios et al. [61] from the same group based on the 2015 ISPD Cardiovascular and Metabolic Guidelines in Adult Peritoneal Dialysis Patients Part I [62] showed that 1-week averaged home SBP is of at least similar accuracy with standardized clinic SBP in diagnosing hypertension confirmed by ambulatory BP monitoring among patients on 84 peritoneal dialysis. In the recent case-control study from this group, 48 male PD patients were matched for age and heart failure status with 48 female patients in a 1:1 ratio [63]. They showed that among PD patients, the levels of ambulatory BP and intensity of antihypertensive treatment were higher in men than in women. However, in these publications, the authors did not focus on ARTH as well as on ISPD guidelines. In the most recent publication, Zhu et al. [50] studied the retrospective cohort of 1442 PD patients from four Chinese dialysis centers. In their study, ATRH was diagnosed in 24.1%, 19.9%, and 24.6%, respectively at 0 years, 3 years, and 1 year after PD start. Patients with ATRH were younger, had a higher BMI, predominantly male, and were smokers, and they also had higher serum creatinine, higher phosphorus, shorter PD vintage, lower Kt/V and lower albumin levels. They more often were treated with RAS inhibitors. There were no differences in residual renal function, ultrafiltration volume, cause of end-stage kidney disease, use of diuretics, or glucose dialysate prescription.

The incidence of ATRH in dialyzed patients differs from one study to another due to the different definitions of treatment used [56,57,64,65]. As in the majority of studies, volume status assessment was not included; therefore, many of the ATRH subjects are misclassified, as that may have hypertension due to volume overload and without resistance to hypotensive therapy [56,57,64,65]. In the setting of loss of RRF and anuria/oliguria, the efficacy of diuretics is limited in dialyzed subjects [24,66]. Some papers stressed the fact that in the setting of limited or no efficacy of diuretic agent in dialyzed patients, the prevalence of ATRH is overestimated when diuretic agents are counted as one class [65]. In our recent review, we presented the limitations and biases of definitions of hypertension, resistant, and apparently resistant hypertension in hemodialysis patients [67]. In our real-life world data, we found a prevalence of ARTH close to 40% in HD. In hemodialyzed subjects, ATRH appears to be multifactorial and influenced rather by factors related more to patients than to dialysis therapy itself. Different ATRH definitions make comparisons between studies difficult [68], particularly with limitations of the available data for analysis such as residual kidney function.

## 6. Managing Resistant Hypertension in ESKD

Resistant hypertension management in ESKD initially did not differ from the general population with hypertension [69,70]. Pseudo-resistant hypertension may be due to medication non-adherence and modification of lifestyle such as increased dietary sodium ingestion, dysfunctional adiposity, or excessive consumption of alcohol, white coat-hypertension, inappropriate technique of BP measurement, marked brachial calcification, intake of various blood pressure-increasing substances/drugs such as various herbs or herbal products, steroids, NSAIDs, erythropoietin/erythropoietin-stimulating agents, alpha mimetics, psychostimulants, illicit drugs, etc. [55]. ABPM should be performed to exclude white coat hypertension, masked hypertension and other forms of spurious resistant hypertension along with non-compliance to non-pharmacological and hypotensive treatment. The most important factors associated with ATRH are hypervolemia and retention of sodium in patients on kidney replacement therapy [19,69].

In hemodialyzed patients, the main target is to achieve dry weight, with the help of dietary sodium restriction, appropriate dialysate sodium prescription, adequate duration of hemodialysis session with appropriate ultrafiltration and adequate intradialytic weight gain not to exceed 1.5 kg between HD sessions [19]. Dry-weight probing, dietary sodium restriction, adjunctive use of loop diuretics, PD regimen adaptation to characteristics of peritoneal transport and the administration of icodextrin solution are the proposed measures to improve the control of BP in PD patients [20] as well as the preservation of RRF and peritoneal membrane function [71]. In patients with high peritoneal transport, a fast switch to APD is a prerequisite. In the setting of loss of residual renal function, hypervolemia and non-compliance, HD should be considered as a viable option. Based on the latest Cochrane meta-analysis [72], the uncertainly of the evidence on APD effect relative to CAPD on overhydration should be underlined as well as blood pressure or dialysis adequacy measures and others. It is due to low or very low certainty evidence. Recently, Dariva et al. [73] in a cohort of PD patients coming from 122 Brazilian dialysis centers (BRAZPD II study) assessed the effect of switching between PD and APD on blood pressure levels. They found that after switching from CAPD to APD, systolic BP decreased by 4 mmHg and increased by 4 mmHg after switching from automated PD to CAPD (*p* = 0.38). The same data were found for diastolic BP without significant changes in the hypotensive medications number started before and after the switch. Similar data were presented Fabian Velasco et al. [74], who showed that transfer from CAPD to APD caused better ultrafiltration, less pronounced edema, decreased mean blood pressure, fewer peritonitis cases and fewer hospitalizations in 300 children. Remote monitoring automated peritoneal dialysis also provides better blood pressure control when compared to CAPD [75].

As shown by Vongsanim and Davenport [76], in a cohort of 510 PD patients (51% on APD with a day-time icodextrin exchange), hypertension was related to elevated NT-proBNP, a marker of extracellular volume expansion and increased peritoneal transport, a positive sodium balance risk factor, and increased urinary sodium suggestive of a higher dietary sodium intake. They postulated sodium restriction and personalized PD prescriptions targeted to control ECW to improve BP control. The use of low-sodium solutions was shown to increase in diffusive peritoneal sodium removal, leading to reduced thirst, lower total body water, and a decrease in nighttime systolic BP [77]. In incremental PD, the appropriate use of icodextrin, minimizing dialysate glucose exposure (avoidance of high glucose dwells), salt restriction, and adequate diuretic use is to be considered to control BP and preserve RRF.

The next step is the intensification of antihypertensive pharmacological therapy. New drugs may help to control BP in ATRH. In hemodialysis patients, sacubitril–valsartan was effective in the therapy of resistant hypertension [78]. In the recent study by He et al. [79] on the sacubitril/valsartan pharmacokinetics and pharmacodynamics properties in 40 peritoneally dialyzed subjects with hypertension or heart failure, mean sitting SBP and mean sitting DBP declined by 19.25 ± 10.32 mmHg and 10.10 ± 8.00 mmHg, respectively, relative to baseline after drug administration. The authors stated that 100 mg of sacubitril/valsartan twice a day was tolerated well and also was safe in this population with good blood pressure control and improvement in cardiac function.

In a small study on 22 PD patients, 50 mg of spironolactone given daily for 1 year seemed to be safe, but there was no significant effect on aortic stiffness [80]. To date, there are no data available on other mineralocorticoid receptor antagonists including finerenone in the PD population.

## 7. Conclusions

Peritoneally dialyzed patients represent a unique population of dialyzed subjects; therefore, studies on the more numerous populations with better quality related to drug adherence and the target blood pressure values should be conducted. The definition of resistant hypertension and apparently resistant hypertension in this group should be revisited, taking into consideration also residual kidney function. Optimal blood pressure management should include appropriate ultrafiltration through either an individual prescription of PD regimen (adequate glucose solution, icodextrin or maybe newer future osmotic solutions), sodium restriction, the judicious use of loop diuretics in nonoliguric patients, and strategies to preserve RRF, which is a vital factor for maintaining volume control and, thereby, control of blood pressure. The issue of compliance is always of utmost importance, making drug pharmacokinetics relevant for the better control of blood pressure, education as well as telemonitoring or other techniques designed to improve adherence/compliance are to be considered for better outcomes.

## 8. Limitations and Future Directions

As PD is less prevalent, data on PD patients are limited or even lacking in some areas including resistant or apparently resistant hypertension. The use of 24 h ABPM is cumbersome in PD patients, as PD is an ambulatory procedure, and patients may live relatively far from the center, making the use of this measurement method logistically challenging. If available, automated office BP measurement with multiple consecutive unattended BP measurements rather than BP readings in routine office assessment may be an option. Similarly to HD, the optimization of volume status to improve blood pressure control is very relevant. The prescription of dialysis and inclusion of icodextrin are to be considered as well as a possible switch to APD [81] or even HD. However, data on the association between optimal blood pressure control, volume status, residual kidney function, dialysis prescription, type of peritoneal membrane and use of icodextrin are lacking. The optimal hypotensive agent is not known in the dialysis population. As the definition of resistant hypertension for general population is not relevant for dialysis setting, therefore, introduction of the term ATRH for subjects who have uncontrolled BP despite the administration of maximally tolerated doses of a beta-blockers, calcium channel blockers, and ACE inhibitors or ARB antagonists should be considered and introduced. Moreover, such subjects require further evaluation for possible other causes and possible therapy with additional hypotensive drugs. In general, the major limitation is the scarcity of data. In addition, some studies are from the last century, and new studies are lacking. Studies on the relationship between apparently resistant hypertension in the PD population and both subclinical and clinical endpoints should be designed, as the issue of resistant or apparently resistant hypertension is still very relevant in clinical practice. Target blood pressure for PD patients is to be established, as guidelines are far from satisfactory.

## Figures and Tables

**Table 1:** Apparent treatment-resistant hypertension (ATRH) prevalence in the recently published studies on PD patients.

Author and Year	Patients	Thresholds of Blood Pressure	ATRH	Assessment of Medication Compliance	Overhydration Systematic Assessment (Method)
Li et al., 2022 [59]	n = 1789 hypertensives Guangdong Province, China	Home BP > 130/80 mmHg Office BP > 140/90 mmHg	42.2% 37.3%	yes subgroup of 15% pts.	no
Vareta et al., 2022 [60]	n = 140 Northern Greece	24 h ABPM > 130/80 mmHg 24 h ABPM > 125/75 mmHg Office BP > 140/90 mmHg Office BP > 130/80 mmHg	30% 32.1% 27.9% 32.9%	no	yes (bioimpedance)
Zhu et al., [50]	n = 1422 4 dialysis centers in southern China	Office BP Systolic BP ≥ 140 mm Hg and/or diastolic BP ≥ 90 mm Hg	24.1%	no	no

## Data Availability

No new data were created.
